# Effects of Visual Attention on Tactile P300 BCI

**DOI:** 10.1155/2020/6549189

**Published:** 2020-02-19

**Authors:** Zongmei Chen, Jing Jin, Ian Daly, Cili Zuo, Xingyu Wang, Andrzej Cichocki

**Affiliations:** ^1^Key Laboratory of Advanced Control and Optimization for Chemical Processes, Ministry of Education, East China University of Science and Technology, Shanghai, China; ^2^Brain-Computer Interfacing and Neural Engineering Laboratory, School of Computer Science and Electronic Engineering, University of Essex, Colchester CO4 3SQ, UK; ^3^Skolkowo Institute of Science and Technology (SKOLTECH), 143026 Moscow, Russia; ^4^Systems Research Institute of Polish Academy of Science, 01-447 Warsaw, Poland; ^5^Department of Informatics, Nicolaus Copernicus University, 87-100 Torun, Poland; ^6^College of Computer Science, Hangzhou Dianzi University, 310018 Hangzhou, China

## Abstract

*Objective*. Tactile P300 brain-computer interfaces (BCIs) can be manipulated by users who only need to focus their attention on a single-target stimulus within a stream of tactile stimuli. To date, a multitude of tactile P300 BCIs have been proposed. In this study, our main purpose is to explore and investigate the effects of visual attention on a tactile P300 BCI. *Approach.* We designed a conventional tactile P300 BCI where vibration stimuli were provided by five stimulators and two of them were fixed on target locations on the participant's left and right wrists. Two conditions (one condition with visual attention and the other condition without visual attention) were tested by eleven healthy participants. *Main Results.* Our results showed that, when participants visually attended to the location of target stimulus, significantly higher classification accuracies and information transfer rates were obtained (both for *p* < 0.05). Furthermore, participants reported that visually attending to the stimulus made it easier to identify the target stimulus in random sequences of vibration stimuli. *Significance*. These findings suggest that visual attention has positive effects on both tactile P300 BCI performance and user-evaluation.

## 1. Introduction

A brain-computer interface (BCI) provides a new pathway between the brain and an external device to achieve direct control and communication [1]. The first BCI system was developed by Vidal in the 1970s [2]. In the decades since, BCIs based on electroencephalography (EEG) recordings have been increasingly frequently explored as they are safe and relatively cheaper than BCIs based on other neuroimaging technologies. The EEG is recorded from sets of electrodes placed on the scalp and comprises a time series of electropotentials generated in the cerebral cortex [3]. The selection of electrode positions and their quantity generally depends on the aims of the study, the ultimate aim of which is, typically, to achieve optimal system performance. The acquired brain signals (e.g., the EEG data) from the selected channels are usually processed through the following steps: preprocessing, feature extraction, feature selection, and classification. These processes seek to identify the intention of the user in order to generate corresponding commands. Finally, these commands can be used for practical applications including, but not limited to, wheelchair navigation [4, 5], character speller [6, 7], and robotic arm manipulation [8, 9].

The brain activities that are most frequently used to control BCI systems include event-related potentials (ERP) [10], steady-state evoked potentials [11], and event-related desynchronization (ERD) [12] and event-related synchronization (ERS) [13]. In an ERP-based BCI, the induction of the ERP is achieved by presenting a predictable sequence of stimuli with one or more rarely, randomly occurring (unpredictable) stimuli interleaved amongst the predictable stimuli. The users are instructed to effectively discriminate the stimuli by means of counting the number of rare stimuli occurrences (presenting at a low frequency and referred to as the “target stimuli”), while ignoring other nontarget stimuli. The P300 is one of the positive components of the ERP and occurs around 300 ms after a target stimulus presentation [14].

Early BCI systems were primarily based on stimuli that were presented visually. For example, the first visual P300 BCI was reported by Farwell and Donchin in 1988 [15] and used a 6 × 6 letters matrix, which was displayed to participants as stimuli on a computer screen. Following on from this work, some researchers took measures to pursue better system performance, an effort which led to, amongst other work, an influential study in which traditional visual letters were replaced with faces [16].

However, the standard visual P300 BCIs depend on gaze control and are not suitable for visually impaired individuals. Consequently, the auditory and tactile P300 BCIs were gradually explored as alternative solutions. Hill et al. [17] first proposed an auditory P300 BCI in which the auditory stimuli were composed of deviant and standard tones. The feasibility of primary tactile P300 BCI was demonstrated by Brouwer and Van Erp [14]. In their study, motors providing vibration stimuli were situated at different locations around the participant's waist. The effects of the number of motors and the stimulus onset asynchrony (SOA) on classification performance were also investigated.

Our study focuses on the tactile P300 BCI. Researchers have attempted to apply tactile stimulation to various parts of body, such as chest [18], fingers [19], back [20], and head [21]. In addition, in order to improve the performance of tactile P300 BCIs, multisensory BCI systems have also been proposed. For example, Brouwer et al. combined tactile stimuli with visual stimuli to construct a visual-tactile bimodal P300 BCI [22], and Yin et al. proposed an auditory-tactile bimodal P300 BCI [23]. Both of them found that BCI with bimodal stimuli obtained higher classification performance compared to that with unimodal stimuli.

In this study, we investigate whether visual attention by the BCI user has any effects on the tactile P300 BCI performance and on the usability of the BCI (as assessed by user-evaluation). A conventional tactile P300 BCI was designed in which vibration stimuli were delivered respectively to participant's left wrist, right wrist, abdomen, left ankle, and right ankle. The participant was asked to distinguish the stimulus on the left wrist or right wrist from other stimuli. Two conditions were tested by participants: one condition used visual attention (called the VA condition) and the other did not use visual attention (called the NVA condition). Notably, in the NVA condition, the participants were required to silently count the number of target vibration appearances only by spatial attention. While in the VA condition, in addition to the counting tasks, the participants also had to pay visual attention to the target vibration location all the time from the short target vibration cue until the target cue moved to another location.

## 2. Materials and Methods

### 2.1. Participants

A total of eleven healthy adults from East China University of Science and Technology in Shanghai, China (including 4 females and 7 males, aged from 22 to 26) participated in this study; they were designated P1, P2,…, P11. All participants had normal or corrected-to-normal vision and intact tactile sensation (self-reported). Importantly, none of them were trained before. In order to achieve the aim of the study, the experimental procedure and the required tasks were explained in detail before any of the individuals participated. Moreover, each participant signed a written consent form prior to the study, which was approved by the local ethics committee.

### 2.2. Stimuli and Procedure

**Figure 1:**
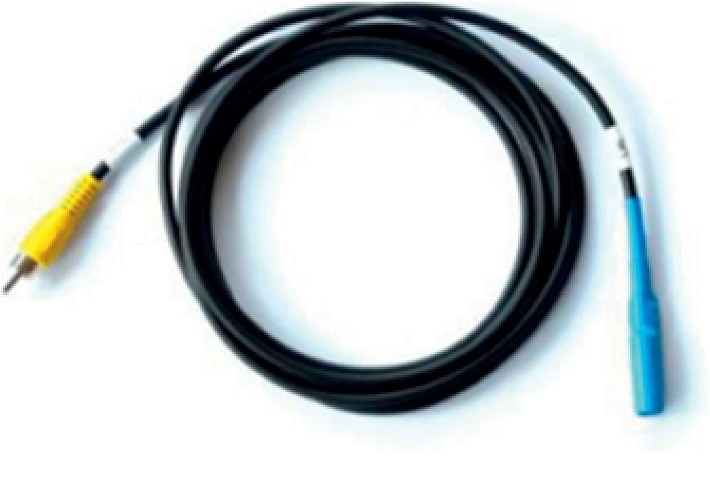
The vibrotactile stimulator.

The vibrotactile stimuli were provided by g.VIBROstims, the main unit of which was DC motors that produced the vibrations. As shown in Figure 1, the motor was hidden in a cylindric casing, which was placed on the participant's body by adhesive plaster. The g.VIBROstims were driven by a g.STIMbox (g.tec medical engineering GmbH, Schiedlberg, Austria), which was connected to the computer via USB and was controlled by a Simulink block (Matlab 2015b). Based on a previous research, the stimulus duration was set to 200 ms and the interstimulus interval (ISI) was set to 400 ms [22].

**Figure 2:**
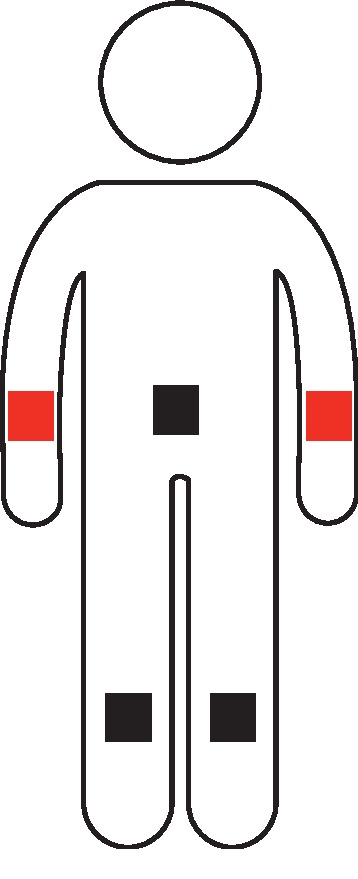
The placements of the vibration stimulators on each participant's body.

For both conditions, each participant sat in a chair in front of a monitor and the vibration stimulators were placed on the left wrist, right wrist, abdomen, left ankle, and right ankle, which ensures sufficient spatial distance to achieve distinguishability between individual stimuli.  Figure 2 shows the placements of vibration stimulators on each participant's body. Compared to the abdomen, left ankle, and right ankle, the left and right wrists are easier for visual attention. In addition, if participants pay visual attention to the abdomen, left ankle, or right ankle, it will bring larger movements of the head or eyeball. So only the left and right wrists were selected as the target stimulus positions where the stimulators were marked in red. The rest of the stimulators were never selected as target stimulus positions and only were used as standard stimulus positions for reducing the probability of the target stimulus presentation, in which the stimulators were marked in black. Each participant's task was to silently count the number of times the target vibration was presented and avoid unnecessary body movements. In particular, for the VA condition, besides the counting tasks, the participants were also asked to give visual attention to the target stimulus positions. The positional conversion of visual attention and the stimulus location that the participant needed to attend to both depended on a particular target vibration cue. To prevent head or eye movement caused by positional shift of visual attention, before carrying out each condition, the participants were asked to place their left and right hands on a desk and their left and right wrists were simultaneously shown in the field of view. Furthermore, the participants were told to immediately switch visual attention in accordance with the target vibration cue. Conversely, for all participants, there was no visual attention to the target stimulus positions during presentation of the NVA condition.

**Figure 3:**
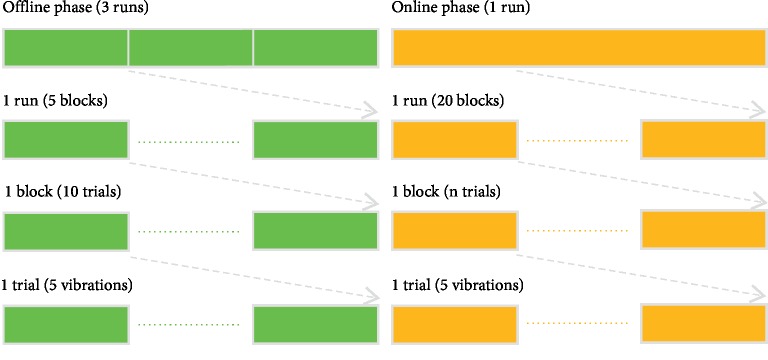
The procedure of each experiment.

Each condition required participants to complete a corresponding experiment, and they should be done on the same day. The order of two experiments was random. In our study, six participants chose to do the VA condition experiment first. In order for the participant to maintain sufficient energy to complete each experiment, there would be an interval between the two experiments, the length of which depended on the individual. Each experiment contained an offline phase and an online phase (see Figure 3). In the offline phase, three runs were included and each run consisted of five blocks (i.e., five target selections). Prior to each block, the target vibration cue was presented for 1.5 s. There were 10 trials per block and all trials within a block had the same target. In each trial, five vibrations occurred randomly. To mitigate for fatigue, each participant could take a short break between offline runs. Furthermore, a long break was used to allow participants to prepare for the following online phase. The length of time of both breaks depended on the individuals. In the online phase, only one run was involved, but there were 20 blocks (i.e., 20 target selections). The number of trials per block (*n*) was variable, which was automatically determined based on an adaptive strategy [24], and each trial also was composed of five vibrations.

### 2.3. EEG Acquisition

**Figure 4:**
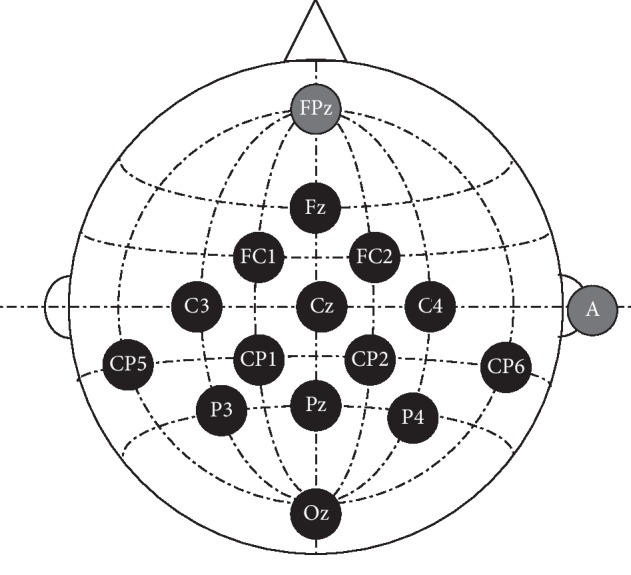
The configuration of all selected electrode positions.

For each participant, EEG data was recorded at a sampling rate of 256 Hz with a g.USBamp (high-pass and low-pass filters set at 0.1 Hz and 30 Hz; a notch filter set at 50 Hz) and a g.EEGcap (Guger Technologies, Graz, Austria). EEG electrodes were positioned according to the international 10-20 system. In our study, fourteen wet active Ag-AgCl electrodes (Fz, FC1, FC2, C3, Cz, C4, CP3, CP1, CP2, CP4, P3, Pz, P4, and Oz) were selected. In addition, FPz was selected as the ground electrode and the right mastoid (A) was selected as the reference electrode. As shown in Figure 4, the black circles mark the 14 EEG recording electrodes, while the gray circles mark the ground electrode (FPz) and reference electrode (A). The impedances of these electrodes were below 10 kΩ and EEG waveforms from all channels remained relatively stable at the start of each experiment.

### 2.4. Feature Extraction and Classification

In each experiment, an 800 ms data segment was extracted after each vibration stimulus presentation. This resulted in a total of 750 data segments, including 150 targets and 600 nontargets, extracted from the offline phase of the experiment. Each EEG data segment was filtered into the frequency range 0.1–30 Hz by a 3^rd^ order Butterworth band-pass filter and then downsampled from 256 Hz to 36.6 Hz by selecting every seventh sample. Therefore, a spatiotemporal feature vector was formed with a dimensionality of 14 × 29 (14 channels and 29 sample points). In this case, 750 such feature vectors were collected as calibration data for each condition. Moreover, winsorizing was adopted to remove interference signals resulting from muscle activity, eye blinks, or eye movement. Firstly, the 10th and 90th percentiles for each sample were computed; secondly, the values of each sample lying less than the 10th percentile or more than the 90th percentile were replaced with the 10th or the 90th percentile, respectively [25, 26].

Bayesian linear discriminant analysis (BLDA) was chosen to build the classifier model for online validation. This approach has been widely employed in an increasing number of P300 BCI systems due to its superior classification performance [27, 28]. The classification rule can be defined as

(1) $$\begin{array}{c} m=\beta {\left(\beta XX^{T}+I^{\prime }\left(\alpha \right)\right)}^{-1}Xt,\\ y=m^{\prime }x, \end{array}$$

where *m* denotes the discriminant vector, two hyper parameters *α* and *β* are the inverse variance of prior distribution and noise, *X* denotes a matrix containing feature vectors, and *t* denotes the regression targets, which is regulated for class 1 in *N*/*N*_1_ and for class 2 in −*N*/*N*_2_ (where *N*_1_ is the number of features from class 1, *N*_2_ is the number of features from class 2, and *N* is the total number of features from both classes). The variable *y* denotes the output of the classifier, and *x* denotes the new input feature vector.

For online classification and recognition in our study, five spatiotemporal feature vectors were obtained from five vibrations (i.e., five stimulus positions) during each single trial. These were then input into the classifier to calculate whether their probability distributions belong to the target class. Finally, the stimulus position with the maximal probability distribution was identified and reported as the classification result.

### 2.5. Performed Analysis

In this paper, in order to investigate whether visual attention had any effects on the tactile P300 BCI performance, we analyzed both the offline and online data recorded during presentation of the VA and NVA conditions. For the offline data recorded during presentation of the VA and NVA conditions, the ERP amplitudes and the *r*-squared values were used to show how ERPs differed between the two conditions. The definition of *r*-squared values is as follows:

(2) $$\begin{array}{c} r^{2}={\left(\frac{\sqrt{N_{1}N_{2}}}{N_{1}+N_{2}}\cdot \frac{\text{mean}\left(X_{1}\right)-\text{mean}\left(X_{2}\right)}{\text{std}\left(X_{1}\cup X_{2}\right)}\right)}^{2}, \end{array}$$

where *N*_*i*_ denotes the features of each class and *X*_*i*_ denotes the number of samples (*i* = 1, 2).

In addition, we explored the amplitude (i.e., the peak value) and latency (i.e., the peak time) of the N200, P300, and N400 ERPs at different electrode sites averaged across 11 participants for each condition. Apart from these, the mean amplitude of the P300 ERP at electrode Cz for each participant was also analyzed. For the purpose of comparing the offline performance differences between the two stimulation conditions, the offline classification accuracy and raw bit rate (i.e., information transfer rate) were both averaged across the 11 participants across 1–10 trials [29], and the offline classification accuracies, based on single trials, for the 11 participants were calculated. We also analyzed the contributions of the N200, P300, and N400 ERPs to the classification accuracy, as well as the single-target classification accuracy for the 11 participants with each stimulation condition. To further make a comparison of the online performance differences between the NVA and VA conditions, based on online data, the online classification accuracy, raw bit rate, and required average number of trials used to classify each position were calculated.

Before carrying out any statistical comparison between data obtained from these two conditions, we first tested the normality of the data (one-sample Kolmogorov–Smirnov tests). For the data that were observed to be normally distributed, we used paired-samples *t*-tests to estimate the significance of the differences, while for the data that was not observed to be normally distributed, a nonparametric test was needed. Therefore, we chose a Wilcoxon signed-rank test to make a comparison [26, 30]. The significance level was set to *p* < 0.05.

### 2.6. Subjective Feedback

The feedback from participants can provide further information that allows us to investigate the effects of visual attention on user-evaluation when using a conventional tactile P300 BCI. Consequently, we conducted a questionnaire survey after each participant completed the corresponding experiments for the two conditions. The questions were delivered in Chinese (the first language of all 11 participants). The English translations of the questions are as follows:

1. Which condition did you feel was more difficult to use? Please give scores to both conditions on a scale of one to five. The higher the score, the more difficult you feel the condition was to use.
2. Which condition made you feel more tired? Please give scores to both conditions on a scale of one to five. The higher the score, the more tired you felt as a result of using this condition.

## 3. Results

### 3.1. ERPs

**Figure 5:**
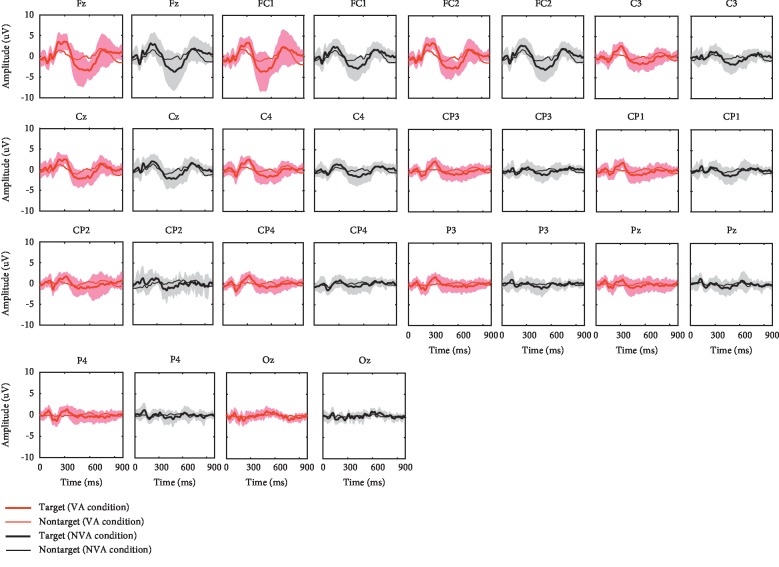
The grand averaged ERPs for the target and nontarget stimuli averaged across all 11 participants for each of the 14 electrode sites.

**Figure 6:**
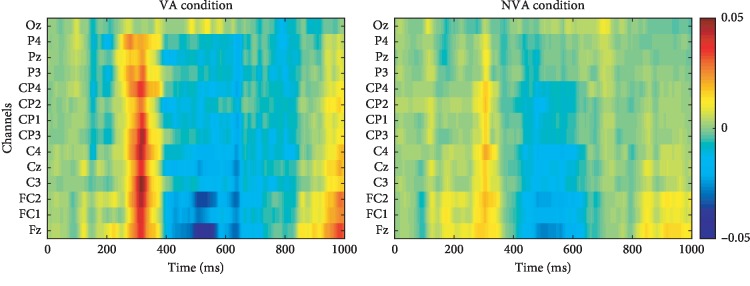
The *r*-squared values of the ERPs from 0 to 1000 ms, averaged over all 11 participants.

Figure 5 shows the grand averaged ERPs when attending to the targets and the nontargets over all 11 participants, for each of the 14 electrode sites.  Figure 6 shows the r-squared values of the ERPs from 0 to 1000 ms, averaged over all 11 participants. It can be observed that the VA and NVA conditions had similar ERP components (see Figure 5), but the feature difference between targets and nontargets in the VA condition was larger than that in the NVA condition (see Figure 6).

Table 1 shows the mean peak values and peak times of the N200, P300, and N400 ERPs at different electrode sites averaged over all 11 participants. For the N200 and P300 ERPs, the most negative peak and the most positive peak were, respectively, observed to occur between the 100–250 ms and 250–400 ms. The most negative peak of the N400 ERP was observed to occur from 400 ms to 650 ms after the stimulus [31]. This result shows that the N200 ERP, recorded from electrode Pz and evoked by the VA stimulation condition, had a higher absolute mean peak value and a shorter peak duration than the ERP evoked by the NVA condition. The same result was observed in the case of the N400 ERP. Similarly, the P300 ERP had a higher absolute mean peak value and a shorter peak duration when recorded from both electrodes Pz and Cz during the VA stimulation condition, compared to the NVA condition.

**Figure 7:**
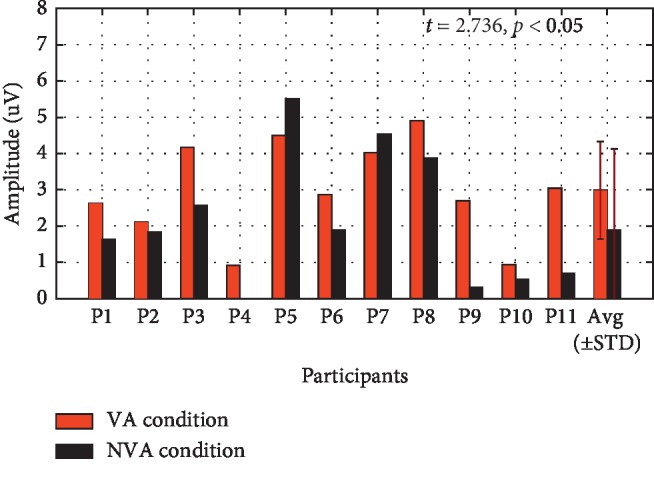
The mean amplitude of the P300 ERP recorded at electrode Cz for each participant.

Figure 7 shows the mean amplitude of the P300 ERP, for each participant, recorded from electrode Cz. The mean amplitude was averaged from each ERP peak point ±25 ms [27, 32]. The result of paired-samples *t*-tests showed that the mean amplitude of the P300 ERP at electrode Cz during presentation of the VA condition was significantly larger than during presentation of the NVA condition (*t* = 2.736, *p* < 0.05)).

### 3.2. Offline Performance

**Figure 8:**
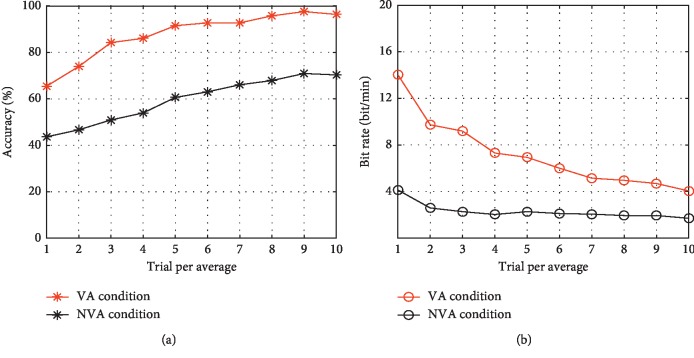
The mean offline performance averaged over all 11 participants across 1–10 trials. (a) The classification accuracy. (b) The raw bit rate.

**Figure 9:**
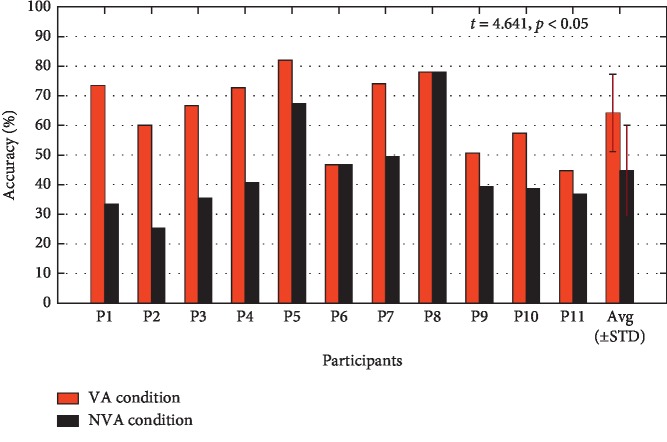
The single-trial classification accuracy for each participant using the offline data for each of the two stimulation conditions.

Figure 8 shows the mean offline performance averaged over all 11 participants across 1–10 trials. The offline classification accuracy (see Figure 8(a)) and raw bit rate (see Figure 8(b)) were calculated from 15-fold cross-validation. The offline classification accuracy and raw bit rate of the VA condition were both significantly higher than those of the NVA condition.  Figure 9 shows the single-trial classification accuracy of each participant using the offline data for each of the two stimulation conditions. The results of paired-samples *t*-tests showed that the VA condition achieved significantly higher single-trial classification accuracy than that achieved with the NVA condition (*t* = 4.641, *p* < 0.05).

**Figure 10:**
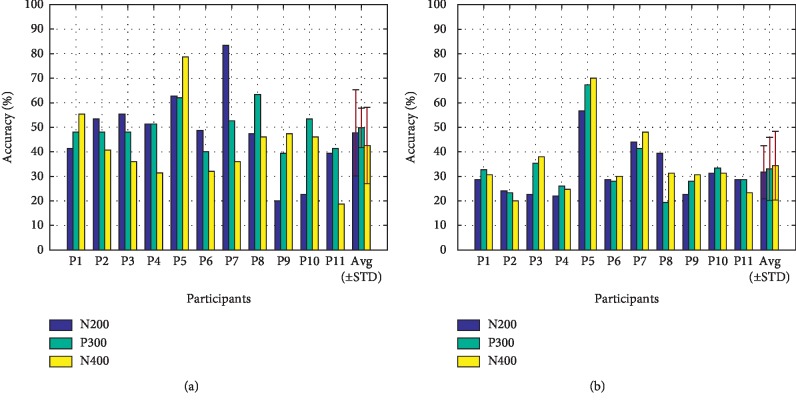
The contributions of N200, P300, and N400 ERPs to the offline classification accuracy for each participant. (a) VA condition. (b) NVA condition.

**Figure 11:**
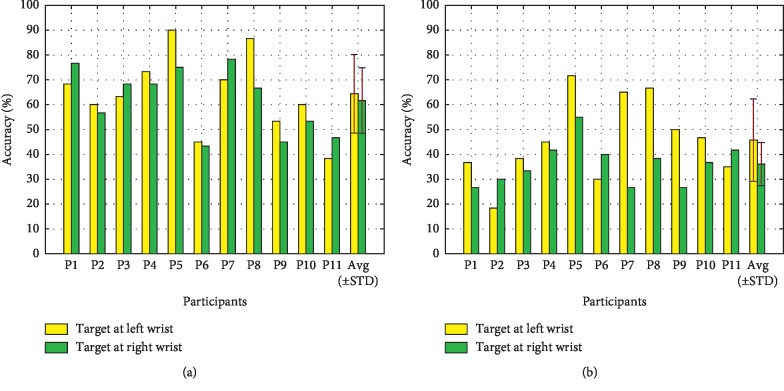
The offline single-target classification accuracy for each participant. (a) VA condition. (b) NVA condition.

Figure 10 shows the contributions of the N200 (peaking between 150 ms and 300 ms), the P300 (300 ms and 450 ms), and the N400 (450 am and 700 ms) ERPs to offline classification accuracy for each participant. It can be seen that all the time windows were crucial in achieving the classification results. The results of paired-samples *t*-tests showed that the contributions of these three ERPs to offline classification accuracy in the VA condition were all significantly higher than those in the NVA condition (N200: *t* = 3.472, *p* < 0.05; P300: *t* = 4.539, *p* < 0.05; N400: *t* = 2.380, *p* < 0.05).  Figure 11 shows the offline single-target classification accuracy for each participant. Most participants achieved higher classification accuracy with the left wrist than that with the right wrist for each stimulation condition (see the left panel of Figure 11, 7 out of 11 participants for the VA condition; see the right panel of Figure 11, 8 out of 11 participants for the NVA condition). The results of paired-samples *t*-tests showed that the VA condition achieved significantly higher single-target classification accuracy than that achieved with the NVA condition (target at left wrist: *t* = 4.993, *p* < 0.05; target at right wrist: *t* = 5.418, *p* < 0.05).

### 3.3. Online Performance

Table 2 shows the online classification accuracy, average number of trials, and raw bit rate for each participant in the two stimulation conditions. The classification accuracy and raw bit rate of the VA condition were significantly higher than those of the NVA condition (*t* = 8.484, *p* < 0.05 for classification accuracy; *t* = 7.667, *p* < 0.05 for raw bit rate). Moreover, the average number of trials of the VA condition was significantly less than that of the NVA condition (*t* = −3.688, *p* < 0.05).

### 3.4. Participant Evaluation

Table 3 shows the scores given by the 11 participants to the two questions for each condition. Compared to the NVA condition, the VA condition obtained lower scores in terms of both the degree of difficulty and the tiredness resulting from using the stimulation condition for all the participants. This demonstrated that all 11 participants felt the NVA condition to be more difficult and tiring than the VA condition. The result of a nonparametric Wilcoxon signed-rank test showed that there were significant differences between the two conditions in both the degree of difficulty (*p* < 0.05) and the degree of tiredness (*p* < 0.05).

## 4. Discussion

In the current study, we designed a conventional tactile P300 BCI, in which five tactile stimulators were spatially distributed over a participant's left wrist, right wrist, abdomen, left ankle, and right ankle. Only the left and right wrists were selected as target stimulus positions and the rest were used as standard stimulus positions. Junichi Hori et al. have reported that the frequency of each stimulus should be consistent to prevent the P300 ERP occurring in response to the nontarget stimuli with some participants [33]. Therefore, the standard stimuli in our study were placed on three different body positions as a solution to this problem. In order to explore whether visual attention had effects on this tactile P300 BCI, the VA and NVA conditions were setup and tested by 11 participants. In each trial of the two conditions, five stimulators vibrated randomly and the participant performed a counting task to count the target stimulus onsets. At this time, the targets and nontargets could be endogenously discriminated based on their location. In contrast to the NVA condition, the participants using the VA condition were also instructed to pay visual attention to the position of the target vibration. It is worth noting that there are only tactile stimuli without visual stimuli in the VA condition, so it is still categorized as a unimodal BCI. It is different from the visual-tactile bimodal BCI designed by Brouwer et al., in which both tactile and visual-tactile stimuli are provided and the visual stimulus reflects the same tap pattern as presented by the tactor [22]. The presentation of visual stimuli requires certain equipment provided externally, and there will be visual potentials in the subject's EEG. However, these will not happen when paying attention to the position of the tactor on the body.

Researches have shown that spatial attention can be used to modulate ERP components [34, 35]. This corresponds to our findings that the N200, P300, and N400 ERPs were evoked during both the VA and NVA conditions (see Figure 5). Specifically, the VA condition yielded more discriminative features between targets and nontargets compared to the NVA condition (see Figure 6). The mean peak values of the N200 ERP at electrode Pz, the P300 ERP at electrodes Pz and Cz, and the N400 ERP at electrode Cz during the VA condition were higher than those observed during the NVA condition. However, the mean peak times of the N200 ERP at electrode Pz, the P300 ERP at electrodes Pz and Cz, and the N400 ERP at electrode Cz during the VA condition were lower than those observed during the NVA condition (see Table 1).

As for the mean amplitude of the P300 ERP at electrode Cz for each participant, a significant difference was observed between the two conditions (see Figure 7). Additionally, the mean offline classification accuracy and raw bit rate over the 11 participants, when different numbers of trials were used to construct the ERP (1–10 trials) during the VA condition, were higher than those observed during the NVA condition in the first trail. Subsequently, the classification accuracy of both conditions improved gradually as the number of trials increased. Finally, both conditions achieved a classification accuracy higher than 70% (see Figure 8(a), 96.36% was achieved for the VA condition; 70.30% for the NVA condition), and this is considered as the minimum accuracy percentage necessary for effective BCI control [36].

When single-trial classification was used, the offline classification accuracy of the VA condition was significantly higher than that of the NVA condition (see Figure 9). For each condition, the contributions of the N200, P300, and N400 ERPs to the offline classification accuracy for each participant were different (see Figure 10), but the contributions of all the time windows to offline classification accuracy in the VA condition were all significantly higher than those in the NVA condition. We found that the late ERPs contributed more to the classification accuracy than the early ERPs for most participants in the VA condition, while the NVA condition happened to be the opposite. In this study, the left and right wrists were used for delivering target vibration stimuli, which allowed for visual attention and discrimination between targets due to their spatial distribution. The resulting single-target classification accuracies showed that the mean classification accuracy of the left target was higher than that of the right target for both of the conditions (see Figure 11). However, the single-target classification accuracy showed that there was no significant difference between two target locations in both paradigms. This phenomenon can be explained by the description in the somatosensory homunculus that the left and right sides of the wrists have similar tactile sensitivity [37]. Significantly higher single-target classification accuracies were achieved with the VA condition than those achieved with the NVA condition.

The online results showed that the classification accuracy and raw bit rate of the VA condition were both significantly higher than those of the NVA condition (see Table 2), which proved that the VA stimulation condition was feasible and effective. In particular, in the VA condition, three participants obtained a peak online classification accuracy of 100% and 8 out of the 11 participants achieved online classification accuracies higher than 90%. Moreover, the lowest online classification accuracy (75%) in the VA condition is equivalent to the highest online classification accuracy in the NVA condition. As in all cases, the VA condition could obtain superior performance compared to the NVA condition.

According to the feedback provided by the 11 participants who attempted to control the BCI using both stimulation conditions, it was easier to clearly count the number of times the target stimuli was presented in the VA condition. Most importantly, all participants hold the view that the NVA condition made them more tired compared to the VA condition (see Table 3). On one hand, these phenomena indicated that visual attention could help the participants pay more attention to the targets and avoid forgetting the position of the target stimuli. On the other hand, in order to accurately count the number of times the target appears, the participants needed to spatially concentrate on the target, which could cause fatigue and discomfort over a prolonged period of time. Conversely, visual attention would deal with these problems and make the participants feel relaxed.

## 5. Conclusions

The main goal of this study was to assess the influence of using visual attention during attempted control of a conventional tactile P300 BCI. Two stimulation conditions were explored and compared. The test results of eleven participants showed that the VA condition could obtain superior performance and was preferred by the participants over the NVA condition. Thus, the involvement of visual attention can have positive effects on both tactile P300 BCI performance and user-evaluation. Future work will concentrate on further optimization such tactile BCI stimulation conditions and on further validation by more participants and BCI end user groups.

## Figures and Tables

**Table 1:** The mean peak values and peak times of the N200, P300, and N400 ERPs at different electrode sites averaged over all 11 participants.

ERP	Peak value (μV)		Peak time (ms)	
	VA condition	NVA condition	VA condition	NVA condition
N200_Pz	−2.34	−2.26	193.89	207.74
P300_Pz	2.12	1.62	341.97	348.37
P300_Cz	3.80	2.77	316.76	332.39
N400_Cz	−5.23	−4.44	535.51	539.06

**Table 2:** The online classification accuracy, average number of trials, and raw bit rate for each participant.

Participants	ACC (%)		AVT		RBR (bit/min)	
	VA-C	NVA-C	VA-C	NVA-C	VA-C	NVA-C
P1	95	65	3.05	3.35	12.69	4.11
P2	90	50	3.15	3.85	10.49	1.67
P3	100	75	3.00	3.15	15.48	6.42
P4	100	55	3.25	3.35	14.29	2.56
P5	100	60	3.05	3.30	15.23	3.34
P6	80	55	3.30	3.50	7.27	2.45
P7	90	55	3.10	3.65	10.66	2.35
P8	95	70	3.05	3.35	12.69	5.02
P9	95	75	3.30	3.25	11.73	6.22
P10	75	65	3.40	3.60	5.95	3.82
P11	80	65	3.35	3.35	7.16	4.11
AVG ± STD	90.91 ± 8.89	62.73 ± 8.47	3.18 ± 0.14	3.43 ± 0.20	11.24 ± 3.30	3.82 ± 1.57

ACC refers to classification accuracy, AVT refers to average number of trials, RBR refers to raw bit rate, VA-C refers to VA condition, NVA-C refers to NVA condition, AVG refers to average, and STD refers to standard deviation.

**Table 3:** The scores given by the 11 participants to the two questions for each stimulation condition.

Participants	Difficult		Tired	
	VA-C	NVA-C	VA-C	NVA-C
P1	2	5	1	3
P2	2	4	1	3
P3	3	4	1	2
P4	3	4	1	3
P5	4	5	1	2
P6	3	4	2	3
P7	4	5	2	4
P8	3	4	1	3
P9	3	4	2	4
P10	4	5	1	2
P11	3	4	2	4
AVG ± STD	3.09 ± 0.70	4.36 ± 0.50	1.36 ± 0.50	3.00 ± 0.77

AV-C refers to VA condition, NVA-C refers to NVA condition, AVG refers to average, and STD refers to standard deviation.

## Data Availability

The data used to support the findings of this study are included within the article.
